# Combined acetyl-11-keto-β-boswellic acid and radiation treatment inhibited glioblastoma tumor cells

**DOI:** 10.1371/journal.pone.0198627

**Published:** 2018-07-03

**Authors:** Sefora Conti, Akiva Vexler, Liat Edry-Botzer, Lital Kalich-Philosoph, Benjamin W. Corn, Natan Shtraus, Yaron Meir, Lior Hagoel, Alexander Shtabsky, Sylvia Marmor, Gideon Earon, Shahar Lev-Ari

**Affiliations:** 1 Laboratory of Herbal Medicine and Cancer Research, Institute of Oncology, Tel-Aviv Medical Center affiliated to the Faculty of Medicine, Tel-Aviv University, Tel-Aviv, Israel; 2 Institute of Radiotherapy, Tel-Aviv Medical Center affiliated to the Faculty of Medicine, Tel-Aviv University, Tel-Aviv, Israel; 3 Pathology Department, Tel-Aviv Medical Center affiliated to the Faculty of Medicine, Tel-Aviv University, Tel-Aviv, Israel; Northern University, UNITED STATES

## Abstract

Glioblastoma multiforme (GBM) is the most common and most aggressive subtype of malignant gliomas. The current standard of care for newly diagnosed GBM patients involves maximal surgical debulking, followed by radiation therapy and temozolomide chemotherapy. Despite the advances in GBM therapy, its outcome remains poor with a median survival of less than two years. This poor outcome is partly due to the ability of GBM tumors to acquire adaptive resistance to therapy and in particular to radiation. One of the mechanisms contributing to GBM tumor progression and resistance is an aberrant activation of NF-ĸB, a family of inducible transcription factors that play a pivotal role in regulation of many immune, inflammatory and carcinogenic responses. Acetyl-11-keto-β-boswellic acid (AKBA) is a pentacyclic terpenoid extracted from the gum Ayurvedic therapeutic plant Boswellia serrata. AKBA is anti-inflammatory agent that exhibits potent cytotoxic activities against various types of tumors including GBM. One of the mechanisms underlying AKBA anti-tumor activity is its ability to modulate the NF-ĸB signaling pathway. The present study investigated *in vitro* and *in vivo* the effect of combining AKBA with ionizing radiation in the treatment of GBM and assessed AKBA anti-tumor activity and radio-enhancing potential. The effect of AKBA and/or radiation on the survival of cultured glioblastoma cancer cells was evaluated by XTT assay. The mode of interaction of treatments tested was calculated using CalcuSyn software. Inducing of apoptosis following AKBA treatment was evaluated using flow cytometry. The effect of combined treatment on the expression of PARP protein was analysed by Western blot assay. Ectopic (subcutaneous) GBM model in nude mice was used for the evaluation of the effect of combined treatment on tumor growth. Immunohistochemical analysis of formalin-fixed paraffin-embedded tumor sections was used to assess treatment-related changes in Ki-67, CD31, p53, Bcl-2 and NF-ĸB-inhibitor IĸB-α. AKBA treatment was found to inhibit the survival of all four tested cell lines in a dose dependent manner. The combined treatment resulted in a more significant inhibitory effect compared to the effect of treatment with radiation alone. A synergistic effect was detected in some of the tested cell lines. Flow cytometric analysis with Annexin V-FITC/PI double staining of AKBA treated cells indicated induction of apoptosis. AKBA apoptotic activity was also confirmed by PARP cleavage detected by Western blot analysis. The combined treatment suppressed tumor growth *in vivo* compared to no treatment and each treatment alone. Immunohistochemical analysis showed anti-angiogenic and anti-proliferative activity of AKBA *in vivo*. It also demonstrated a decrease in p53 nuclear staining and in Bcl-2 staining and an increase in IĸB-α staining following AKBA treatment both alone and in combination with radiotherapy. In this study, we demonstrated that AKBA exerts potent anti-proliferative and apoptotic activity, and significantly inhibits both the survival of glioblastoma cells *in vitro* and the growth of tumors generated by these cells. Combination of AKBA with radiotherapy was found to inhibit factors which involved in cell death regulation, tumor progression and radioresistence, therefore it may serve as a novel approach for GBM patients.

## Introduction

Glioblastoma multiforme (GBM) is a particularly aggressive subtype of malignant glioma and the most common and lethal cancer of the central nervous system in adults. GBM is classified as grade IV and it is associated with very poor prognosis. Upon initial diagnosis, the majority of GBM patients, particularly those older than 45 years of age, do not survive longer than one year [1]. The current standard treatment for newly diagnosed GBM patients involves maximal feasible surgical debulking, followed by radiation therapy and concurrent/adjuvant use of temozolomide, an alkylating cytotoxic agent administered for at least 6 months following the end of radiation treatment [2]. Conventional radiotherapy consists of 60 Gy fractionated focal irradiation delivered in daily dose of 2 Gy. The contribution of radiotherapy to standard care of GBM patients has been axiomatic for years, given the increased survival from a range of 3 to 4 months in patients receiving surgery only to a range of 7 to 12 months in patients receiving surgery and radiotherapy [3]. Nevertheless, the outcome of standard treatments for GBM remains poor. Therefore, new approaches are needed to improve the effectiveness of treatment for glioblastoma.

Acetyl-11-keto-β-boswellic acid (AKBA), a pentacyclic terpenoid extracted from the gum of the Ayurvedic therapeutic plant *Boswellia serrata* [4,5] is anti-inflammatory agent that exhibits potent cytotoxic activities against cultured human cancer cells, such as glioblastoma [6], meningioma [7], leukemia [8], breast [9], liver [10], fibrosarcoma, melanoma [11], colon [12], prostate [13] and pancreatic cancer cells [14]. Several *in vivo* studies have also confirmed that AKBA possesses anti-tumor properties [13,15]. AKBA cytotoxic activity has been attributed to its ability to modulate multiple signaling pathways, including NF-ĸB.

Rel/NF-ĸB proteins are a family of inducible transcription factors that play a pivotal role in the regulation of many immune, inflammatory and carcinogenic responses. Aberrant or constitutive activation of NF-ĸB has been linked to tumor promotion in various types of cancer. In fact, NF-ĸB regulates the transcription of many genes involved in cell proliferation, angiogenesis, metastasis, anti-apoptotic responses and resistance to chemotherapeutic drugs and radiation. Several reports have shown a link between NF-ĸB activation and the acquisition of adaptive radioresistance [16–19]. As such, inhibition of NF-ĸB activation could provide a novel approach to enhance the radio-sensitivity and counteract the acquisition of adaptive resistance.

Activation of NF-ĸB has also been linked to the pathogenesis of glioma [20]. A recent study reported a correlation between the over-expression of a p65 subunit of NF-ĸB and the histological grade of the glioma [21]. Another report showed an increased level of phosphorylation of a p65 subunit in GBM compared to normal brain tissue [22]. The two major mechanisms underlying NF-ĸB activation in GBM are thought to be EGFR gene amplification and deletion of the NFKBIA gene that encodes IĸB-α. Amplification and mutations of the EGFR gene are detected in 40–50% of GBMs, whereas the rate of NFKBIA deletions is 23.4% [21].

Several reports have attributed the AKBA anti-tumor effect to its ability to modulate the NF-ĸB pathway. AKBA inhibited proliferation and elicited cell death through suppression of IKK activity in prostate cancer cells [23]. AKBA was also found to potentiate apoptosis, inhibit invasion, and abolish osteoclastogenesis by suppressing NF-ĸB- and NF-ĸB-regulated gene expression [24].

To date, there have been no reports on the efficacy of combining AKBA with radiation as a potential novel approach to enhance the effectiveness of conventional malignant glioma therapy. The aim of the present study was to evaluate the effect of combining AKBA with ionizing radiation in the treatment of glioblastoma tumors and to explore the mechanisms activated by the combined treatment.

## Materials and methods

### Reagents

Acetyl-11-keto-β-boswellic acid—AKBA (Santa Cruz, CA, USA) is characterized by the following properties (according to manufacturer): molecular formula C_32_H_48_O_5_, MW 512.72, purity (HPLC) 99.6% and purity (TLC) 99.6%. AKBA was prepared in stocks of 200 mM in dimethyl sulfoxide (DMSO), stored at -20°C and was thawed and diluted in a cell culture medium immediately before treatment. Antibiotics (penicillin, streptomycin, amphotericin) and a kit for colorimetric tetrazolium salt assay (XTT) for cell survival were obtained from Biological Industries (Beit-Haemek, Israel). Dulbecco’s modified Eagle’s medium (DMEM), and fetal bovine serum (FBS) were purchased from Life Technologies (Rehovot, Israel). Antibodies against PARP, NF-ĸB, p65, IĸB-α, and β-actin were obtained from Santa Cruz Biotechnology (Santa Cruz, CA, USA). Fluorescein isothiocyanate-conjugated annexin V (Annexin V-FITC) with propidium iodide (PI) was received as a Tali apoptosis kit (Life Technologies, Rehovot, Israel).

### Cell lines

Glioblastoma cell lines (astrocytoma grade IV)—U87, U251, A172 were obtained from the American Type Culture Collection and the anaplastic astrocytoma grade III cell line—LN319 was obtained from Da-Ta Biotech. All cell lines were maintained in DMEM supplemented with 10% heat inactivated FBS and 1% penicillin-streptomycin-amphotericin B solution. The cells were cultured at 37°C in a humidified atmosphere of 95% air and 5% CO_2_.

### Irradiation of cells *in vitro*

The cells plated in 96 microwell plates were irradiated with a single 2–6 Gray (Gy) dose 30 min after adding AKBA. A linear accelerator was operated at a 6 mega-electron-volts photon beam at a dose rate of 418 cGy/min. The sample anterior distance was 100 cm and a bolus gel layer (1 cm thick) was placed above the plates.

### Colorimetric tetrazolium salt (XTT) assay for cell survival

The cells (U87,U251, LN319 and A172) were seeded at 1.5-2x10^3^ cells per well in 96-well plates and allowed to attach overnight before being treated with AKBA and/or irradiated with a single dose of ionizing radiation. Cell viability was evaluated using 2,3-Bis-(2-methoxy-4-nitro-5-sulfophenyl)-2H-tetrazolium-5-carboxanilide salt (XTT)-based cell proliferation assay (Biological Industries, Beit-Haemek, Israel). After 72 hrs of incubation, the cells were incubated for 1–3 hrs with XTT originally synthesized by Paull and colleague [25]. Bioreduction of XTT by dehydrogenase enzymes of metabolically active cells yields a highly colored formazan product which was measured at 450 nm wavelength (Sunrise^TM^ absorbance plate reader, Tecan Group AG, Männedorf, Switzerland). Each plate included appropriate blank wells containing media and XTT (but no cells) as well as the control wells containing non-treated cells and only fresh medium. Each variant of the experiment was performed in triplicates and repeated at least twice.

### Isobologram analysis of mode of interaction of treatments tested

The mode of interaction between radiation and AKBA was analyzed using “CalcuSyn” software program (Biosoft, Ferguson, MO, USA and Cambridge, UK) based on Chou and Talalay's equation for calculation of the combination index (CI) [26]. The dose effect curves, normalized isobolograms and CI were determined. A CI of <1.0 indicates synergism of the tested treatments whereas a CI = 1.0 indicates additive activity of the combined treatment.

### Annexin V-FITC/PI double staining assay for apoptosis

The cells (5x10^5^ cells/well) were plated in 6-well plates and treated with AKBA. The adherent and non-adherent cells were collected and double-stained with Annexin V-FITC and PI using a Tali apoptosis kit (Life Technologies, Rehovot, Israel) according to the manufacturer's instructions. Annexin-V-FITC positive cells indicate the early apoptotic population, double positive cells indicate late apoptotic population and PI positive cells indicate the necrotic population. The percentage of apoptotic cells was determined by flow cytometric analysis using a FACS Calibur/Arya instrument (BD Bioscience, San Jose, CA, USA). Data analysis was performed using FlowJo software (Tree Star, Inc., Oregon, USA). Each variant of the experiment was done in triplicates and the experiments were repeated at least twice. The averaged data were calculated and graphed.

### Western blot analysis of protein expression

The cells were cultured at a seeding density of 3-5x10^5^ cells in 60-mm plates and maintained at 37°C in 5% CO_2_ humidified atmosphere. The cells were irradiated and/or treated with AKBA, then harvested and homogenized in lysis buffer [50 mM Tris–HCl, pH 7.4, 0.5% Triton X-100, 150 mM NaCl, 0.1 mM phenyl-methanesulfonyl-fluoride (PMSF) and complete protease inhibitor cocktail] for 30 min on ice. The lysed cells were centrifuged at 13000 g at 4°C for 15 min and the supernatant was collected. Protein concentration was determined using a Bradford/Bio-Rad Protein Assay (Bio-Rad, Hercules, CA, USA; see below). The supernatants (30–50 mg of protein) were separated on a 12% sodium dodecyl sulfate (SDS) polyacrylamide gel and transferred onto a nitrocellulose membrane. After blocking with 5% non-fat milk in PBS buffer containing 0.1% Tween 20 (PBST), the membranes were incubated for 1 hr at room temperature with primary antibody against PARP (1:1000), β-actin (1:1000) and also incubated with HRP-labeled goat anti-rabbit IgG (1:1000) for 1 hr. All blots were detected using SuperSignal West Pico Chemiluminescent Substrate (ThermoScientific, Waltham, MA, USA) and band density was evaluated using ImageJ software.

### Bradford/Bio-Rad Protein Assay

Bovine serum albumin (BSA: 1–10 μg) or cell lysate samples were added in triplicates to wells in a 96-well plate and brought to 160 μl/well with autoclaved deionized filtered water. Forty μl Bio-Rad Quick Start Bradford Dye Reagent (cat # 500–0205) was added and the plate was gently agitated on a plate shaker for 15 min at room temperature (RT). The optical density at 595 nm was determined using a microplate reader (Sunrise^TM^ absorbance plate reader, Tecan Group AG, Männedorf, Switzerland). Protein concentration was determined based on the BSA standard curve.

### Statistics

The *in vitro* experiments were typically performed in triplicates and repeated 2–4 times. The mean values and standard errors were calculated for each point from the pooled normalized data. The significance of difference between the arms was analyzed using the one-tailed Student t-test with unequal variance and was considered as statistically significant if P < 0.05. Efficacy of treatment tested was determined using a non-linear regression model of one-phase decay, and IC50 was defined as the concentration at which 50% of cell killing was obtained.

### *In vivo* studies

Female athymic CD-1 nude mice, 6 to 8 weeks old, were obtained from the Harlan Animal Production Area (Rehovot, Israel). The mice were housed in a laminar air flow cabinet under pathogen-free conditions in standard vinyl cages with air filter tops. Cages, bedding, and water were autoclaved before use. All facilities were approved by the Ethics Committee for Accreditation of Laboratory Animal Care in accordance with the current regulations and standards of the Israeli Ministry of Health. In accordance with the Ethics Committee regulations, the experiment was stopped when the mice became moribund, when the tumors reached 2 cm in their widest diameter, or when the animal’s body weight decreased on 20% of its initial weight. The mice were sacrificed by a lethal dose of CO_2_.

### GBM subcutaneous (s.c.) tumor model in nude mice

To investigate the anti-tumor activity of AKBA alone and combined with radiation, we developed an ectopic (subcutaneous) GBM model in nude mice. This model allows to reveal at the beginning of the experiment tumor bearing mice and only such mice to distribute into different treatment groups and then to follow up tumor growth in each specific marked mouse during the total treatment period. Moreover this model allows to determine the end point of the experiment. In the future we will use the more advanced but more complicated intracranial model.

Using s.c. model we evaluated which cell line tested *in vitro* was most suitable for the study of GBM tumors. Despite the remarkable synergistic effect obtained with A172 cells *in vitro* study, this cell line was not found to be tumorigenic in immune-suppressed mice. In contrast, implantation of U87 and U251 glioblastoma cell lines (10^6^ cells per mouse) was sufficient to develop subcutaneous tumors in all mice. LN319 cell line, even though tumorigenic, is derived from a lower grade astrocytoma (grade III) compared to glioblastoma and therefore was discarded from the *in vivo* part of the present study.

Both generated tumors grew fast, but the growth rate of U87 tumors was significantly higher compared to U251 tumors. H&E staining of histologic sections from generated tumors revealed the invasive nature of the tumor, with cancer cells infiltrating in the muscle tissue. Moreover, H&E staining showed regions with characteristic GBM features: marked cellular atypia, dense cellularity, necrosis with pseudo-palisading, mitotic figures and vascular proliferation.

By establishing tumorigenicity and growth rate of both GBM tumor models, we based our cell line choice on studies concerning genetic and pathological profiles of these two cell lines. The U251 tumor model demonstrated histological and immunohistochemical features of human GBM. In addition, a number of genetic alterations show similarities to human GBM, including alterations in oncogenic pathways and key tumor suppressors such as p53. In contrast, the U87 GBM tumor model shows significant dissimilarities when compared with human GBM, but has nevertheless received significant attention, especially for assessing tumor angiogenesis and anti-angiogenic therapies. In view of the above, U251 cell line was chosen for the *in vivo* study of efficacy and mode of interaction of treatments tested.

To generate GBM tumor, U251 cells were harvested from log-phase cultures by brief trypsinization, then washed once with phosphate-buffered solution (PBS) and suspended at calculated concentrations in PBS. Then, 200 μl inoculums were injected subcutaneously into the flank area of each mouse. Starting from the 7^th^ day after cell transplantation when tumors became palpable, tumor size in each mouse was evaluated twice weekly using a digital caliper: the greatest longitudinal diameter (length) and the greatest transverse diameter (width) were measured. Based on these measurements, tumor volume was calculated by a modified ellipsoidal formula, as follows [27–29]:

$${Tumor\;volume\;(mm}^{3})=[lenght\;(mm)\times {\left(width\;(mm)\right)}^{2}]\times \frac{\pi }{6}$$

Tumor-bearing mice were randomized into the following groups (six mice in each): one control group that received saline treatment, two groups that received only AKBA treatment (20 μg/g and 25 μg/g intra-peritoneal injections twice per week), one group that was irradiated with a single dose (4 Gy) at the beginning of the experiments and one group that received combined treatment (20 μg/g AKBA twice weekly and a single dose of 4 Gy).

### Irradiation of tumor bearing mice

The anesthetized tumor bearing mice were placed in sterile aerated specially designed boxes and irradiated locally with a single dose of 4 Gy 30 min after the first injection of AKBA. The irradiation was done by a linear accelerator operated at a 6 mega-electron-volts photon beam at a dose rate of 418 cGy/min. The sample anterior distance was 100 cm, and a bolus gel layer (1-cm thick) was placed above the mice. The radiation field was narrowed to include only the body part containing tumor.

### Pathology and immunohistochemistry of developed tumors

On day 35 after the beginning of treatment, all the mice were sacrificed, ending the experiment. The tumors underwent pathologic and immunohistochemical evaluation by two specialists—Dr. Sylvia Marmor and Dr. Alexander Shtabsky at the Pathology Department of Tel-Aviv Sourasky Medical Center. Tissue samples containing tumors were formalin-fixed and embedded in paraffin for routine hematoxylin and eosin (H&E) staining and immunohistochemical staining for CD31, Ki67, IĸB-α, clone DO7 of p53 (mutant and wild-type) and Bcl-2. Antigen retrieval was performed at 95°C in citrate buffer pH = 6.0, 6.4 M sodium citrate dehydrate, 1.6 M citric acid monohydrate for 40 min. The slides were cooled at room temperature for 20 min and washed 3 times for 3 min with Tris-buffer pH 7.6, 0.15 M sodium chloride, 0.05 M Trizma HU. The slides were peroxidase blocked for 5 min, washed as above and then incubated for 30 min with the primary antibody, followed by the secondary antibody (visualization reagent) followed by the substrate-chromogen solution (3,3-diaminobenzidine), and finally counter-stained with hematoxylin. All sections were dehydrated, mounted with coverslips and viewed under a light microscope (Eclipse TS100, Nikon, Japan). Staining was quantified subjectively between 0 and 3+ according to the Dako Co. instructions. The results for the *in vivo* experiments were analyzed by one-way analysis of variance. Statistical significance (*P*<0.05) was established by the post hoc Tukey’s pair wise comparison.

## Results and discussion

### Combined effect of AKBA and radiation on survival of glioblastoma cell lines

The combined effect of AKBA and ionizing radiation on the viability of U87, U251, LN319 and A172 glioblastoma cells was evaluated. The cells were irradiated with a single dose (2, 4 or 6 Gy) and then exposed for 72 hrs to different concentrations of AKBA (10–40 μM) selected according to what was previously reported in literature: for example, Kruger et al. [30] used 10–40 μM of AKBA with hepatocytes cells, and Toden et al. [31] used 7.5–30 μM with colorectal cancer cells. Cell survival was evaluated by XTT colorimetric assay. Each graph represented the average survival of treated cells relative to the survival of untreated control cells. The data are mean ± SE values from three individual experiments, each performed in triplicates. Statistical significance was determined by one way ANOVA test (*P < 0.05; **P < 0.01; ***P < 0.001). Normalized isobolograms indicated the mode of interaction between AKBA and ionizing radiation. Combination index for each variant of the combined treatment was calculated using CalcuSyn software as described in the material and methods section. The thick line is the line of additivity; the data below the line presented synergistic interactions while the data above the line presented sub-additive interaction.

**Fig 1:**
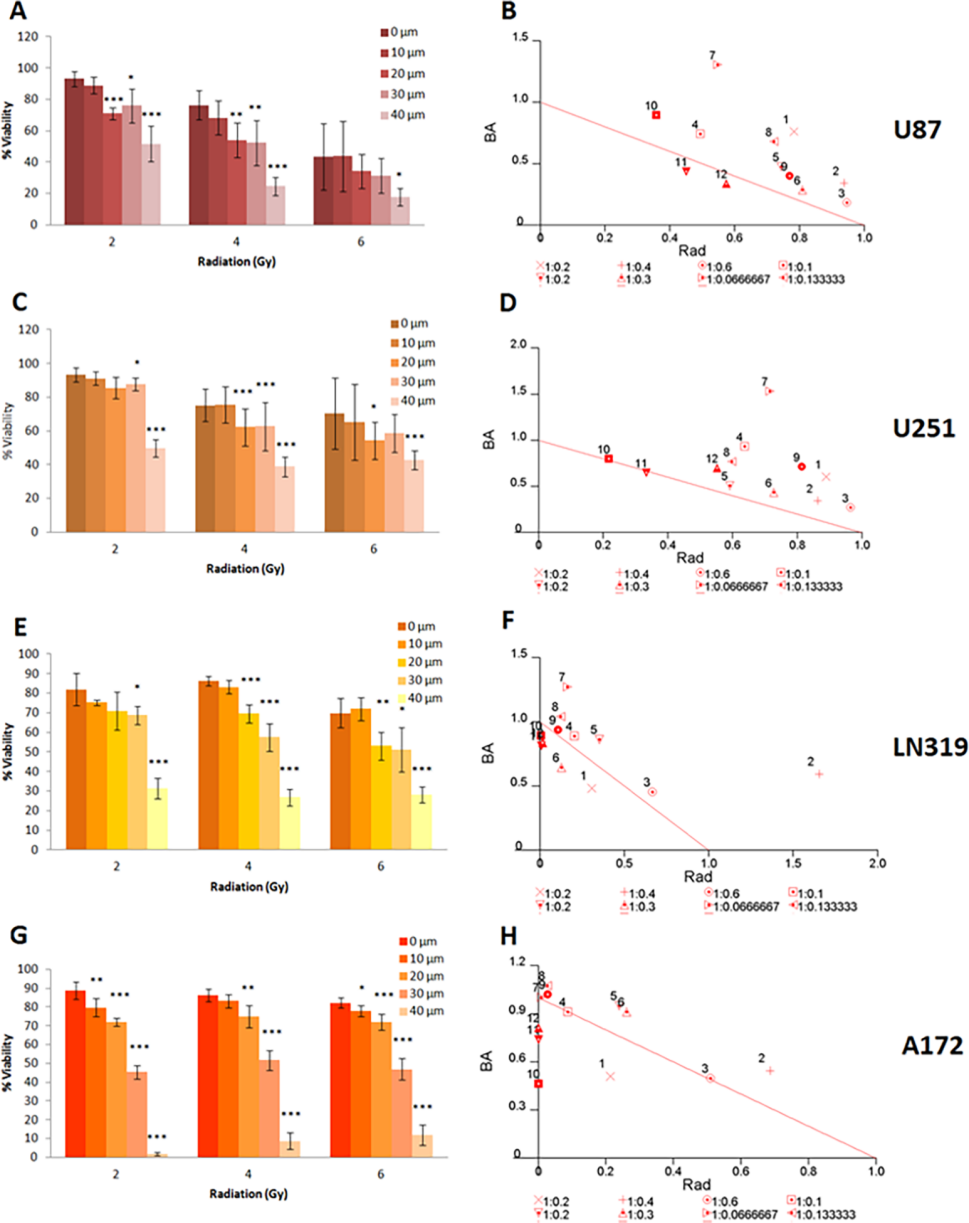
Combined effect of AKBA and ionizing radiation on the survival of glioblastoma cells. Cells were irradiated with a single dose and then exposed for 72 hrs to different concentrations of AKBA. The survival was evaluated by XTT colorimetric assay. The graph represents the average survival of treated cells relative to the survival of untreated control cells. The data are mean ± SE values from three individual experiments, each performed in triplicates. Statistical significance was determined by one way ANOVA test (*P < 0.05; **P < 0.01; ***P < 0.001). Normalized isobolograms indicated the mode of interaction between AKBA and ionizing radiation. Combination index for each variant of the combined treatment was calculated using Calcusyn software. The line is the line of additivity: interactions below the line are synergistic while interactions above the line are sub-additive.

AKBA treatment was found to inhibit the survival of all four tested cell lines in a dose dependent manner (Fig 1). A172 and LN319 cells were more susceptible to the treatment compared to U87 and U251 cells. On A172, the most sensitive cell line, AKBA exerted a significant survival reduction (more than 50%) already at a concentration of 30 μM. In the other three cell lines the same inhibitory effect was obtained at the higher AKBA concentration of 40 μM. At the highest concentration (50 μM) the treatment resulted in less than 3% survival of A172 cells. The most resistant to AKBA cell line was U251 with 20% survival at the highest AKBA concentration (50 μM).

The comparison of sensitivity of glioblastoma cancer cells tested to AKBA and radiotherapy (Table 1) revealed that A172 cell line was the most radio-resistant cell line while it was the most sensitive one to AKBA treatment. In contrast, U87 cell line was AKBA resistant and high sensitive to radiation cell line. LN319 and U251 cell lines demonstrated a moderate sensitivity to both agents tested. As shown in Fig 1, the radiation and AKBA combined treatment resulted in a more significant inhibitory effect compared to treatment with radiation only. To evaluate the mode of interaction between the treatments tested, data were analyzed by CalcuSyn software (Fig 1 and Table 2). Normalized isobolograms obtained from this analysis revealed that the combination of AKBA and radiotherapy resulted in a synergistic effect on A172 cells at high dose (40 μM) and additive effect at lower doses of AKBA. On LN319 and U87 cells the combined effect was strong additive at high dose of AKBA (40 μM).

**Table 1:** Sensitivity of glioblastoma cell lines to treatments tested and respective IC_50_.

Cell line	Effect of AKBA (40 μM)	IC_50_	Effect of radiation (6 Gy)
U87	+	37.02 μM	+++
U251	++	39.73 μM	++
LN319	++	32.02 μM	++
A172	+++	28.63 μM	+

**Table 2:** CalcuSin analysis of AKBA and radiation combined treatment of GBM cells.

AKBA (μM)	Radiation (Gy)	Combination Index (CI)			
		U87	U251	LN319	A172
10	2	1.546	1.495	0.793	0.726
10	4	1.281	1.206	2.246	1.232
10	6	1.13	1.236	1.119	1.007
20	2	1.237	1.571	1.093	1.001
20	4	1.22	1.101	1.215	1.19
20	6	1.098	1.163	0.773	1.175
30	2	1.855	2.248	1.435	1.012
30	4	1.404	1.368	1.166	1.104
30	6	1.172	1.528	1.046	1.05
40	2	1.255	1.017	**0.900**	**0.464**
40	4	**0.888**	0.981	**0.824**	**0.741**
40	6	**0.914**	1.253	**0.852**	**0.813**

### AKBA induced apoptosis correlated with polyADPribose polymerase (PARP) cleavage

Results from the analysis of cell viability following the treatments tested indicated that A172 cell line was the most resistant to ionizing radiation. Moreover, we found that the combined effect on this cell line is the most synergistic among the cell lines tested. Therefore we used A172 cells to test the effect of AKBA and/or radiation on the expression of cell signalling proteins relevant in the quest of elucidating the mechanisms underlying the observed synergistic effect.

**Fig 2:**
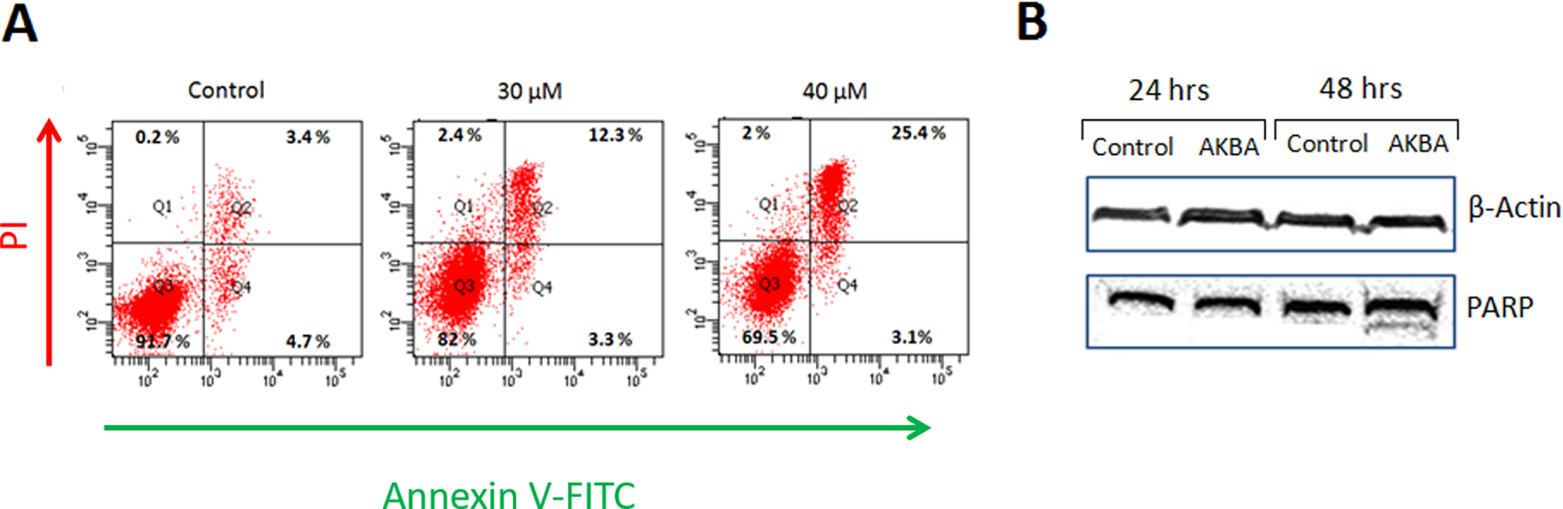
Effect of AKBA on induction of apoptosis in A172 cells. (A) A172 cells were treated with AKBA for 72 hrs and apoptosis was assessed using Annexin-V-FITC/PI double staining and flow cytometry. Double negative cells indicated the live cell population, Annexin-V-FITC positive cells indicated the early apoptotic population, double positive cells indicated late apoptotic population and PI positive cells indicated the necrotic population. (B) Cells were treated with 30 μM AKBA for 24 and 48 hrs. Cell lysates from A172 monolayers were subjected to Western blot analysis with mouse anti-human PARP and β-actin antibodies.

To elucidate the mechanism of AKBA anti-proliferative activity, the induction of apoptosis was examined using Annexin V-FITC/PI double staining and FACS analysis (Fig 2A). Treatment of A172 cells with 30 μM AKBA resulted in 12% apoptotic cells compared to 3% in untreated cells. Moreover, treatment with 40 μM of AKBA induced late apoptosis (double stained cells) in 25% of the cells. Thus, AKBA induces apoptosis in A172 cells in a dose dependent manner.

Western blot analysis was performed to evaluate the expression of PARP following AKBA treatment (Fig 2B). The bands densitometry revealed that treatment of the A172 glioblastoma cell line with AKBA for 48 hrs led to high increase of PARP expression (band density normalized to actin increased from 0.8 to 1.17). Moreover PARP cleavage into a 85 kDa fragments was obvious. But following 24 hrs treatment there was not any changes in PARP expression.

### AKBA and radiation inhibited growth of tumors generated by U251 glioblastoma cells

**Fig 3:**
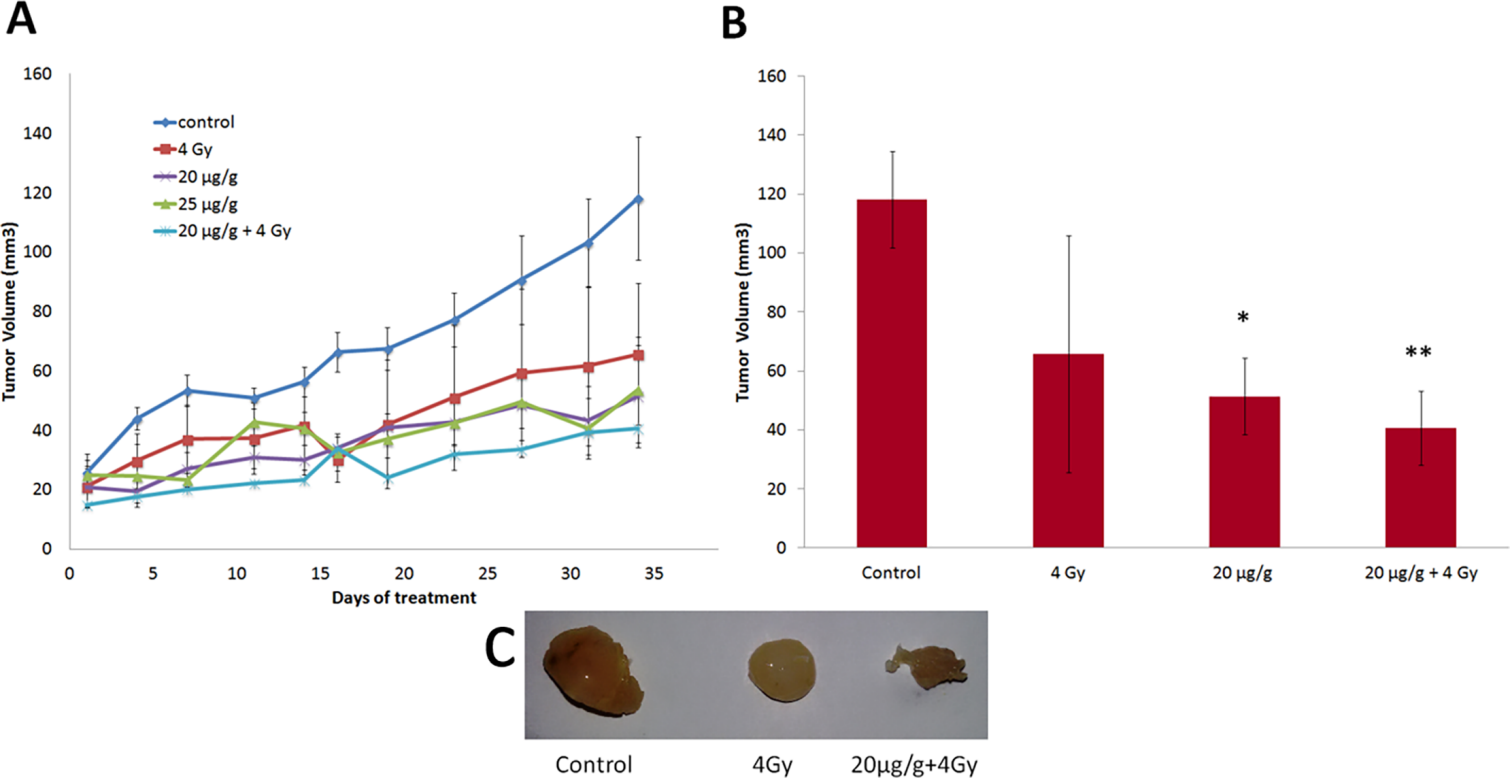
Effect of AKBA, radiation and combined treatment on tumor growth in an ectopic GBM model. **(A)**Tumor growth monitored twice weekly for 7 weeks. Data are mean ± SE values from 6 individual mice in each group. **(B)** The tumor volumes measured at the end of the study, before the mice were sacrificed. The data are averages relative to control with SE (error bars) from 6 individual mice in each group. Differences in tumor volume after exposure to various treatments were determined using the one-way ANOVA test. *P < 0.05; **P< 0.01 vs. controls. **(C)** Representative tumors from corresponding treatments groups.

The s.c. implantation of U251 cells resulted in a generation of tumors that underwent a gradual increase in tumor volume (Fig 3). After 7 days the tumor bearing mice were selected and divided on following treatment groups (6 mice in each): one control group that received saline treatment, two groups that received only AKBA treatment (20 μg/g and 25 μg/g intra-peritoneal injections twice per week), one group that was irradiated with a single dose (4 Gy) at the beginning of the experiments and one group that received combined treatment (20 μg/g AKBA twice weekly and a single dose of 4 Gy).

AKBA was administered intra-peritoneal twice per week. Mice were irradiated with a single dose of 4 Gy at the beginning of AKBA treatment. Tumor size was measured twice per week using digital calliper. The growth rate in the control group was substantially higher compared with the treatment groups. One-way analysis of variance of mean tumor volumes showed a significant difference between the control group (118±21 mm^3^) and the group that received only 20μg/g AKBA (53±18 mm^3^, *P* = 0.018) or a single dose of local irradiation (65 ± 24 mm^3^, *P* = 0.098) (Fig 3A).

The combined AKBA and radiation treatment suppressed tumor growth more significantly (41±10 mm^3^, *P* = 0.002) compared to the growth of untreated tumors (Fig 3).

But the difference between the tumor volume from the groups that received only AKBA treatment or only radiation and the combined treatment group was obvious but not statistically significant (P = 0.547 and *P* = 0.298, respectively).

The tumor samples underwent pathologic and immunohistochemical evaluation at the Pathology Department of Tel-Aviv Sourasky Medical Center. Staining was quantified subjectively between 0 and 3+ according to the Dako Co. instructions by two experts—Dr. Alexander Shtabsky and Dr. Sylvia Marmor, both were blinded to the experimental groups during the analysis.

### Anti-angiogenic and anti-proliferative AKBA activity in GBM tumors

**Fig 4:**
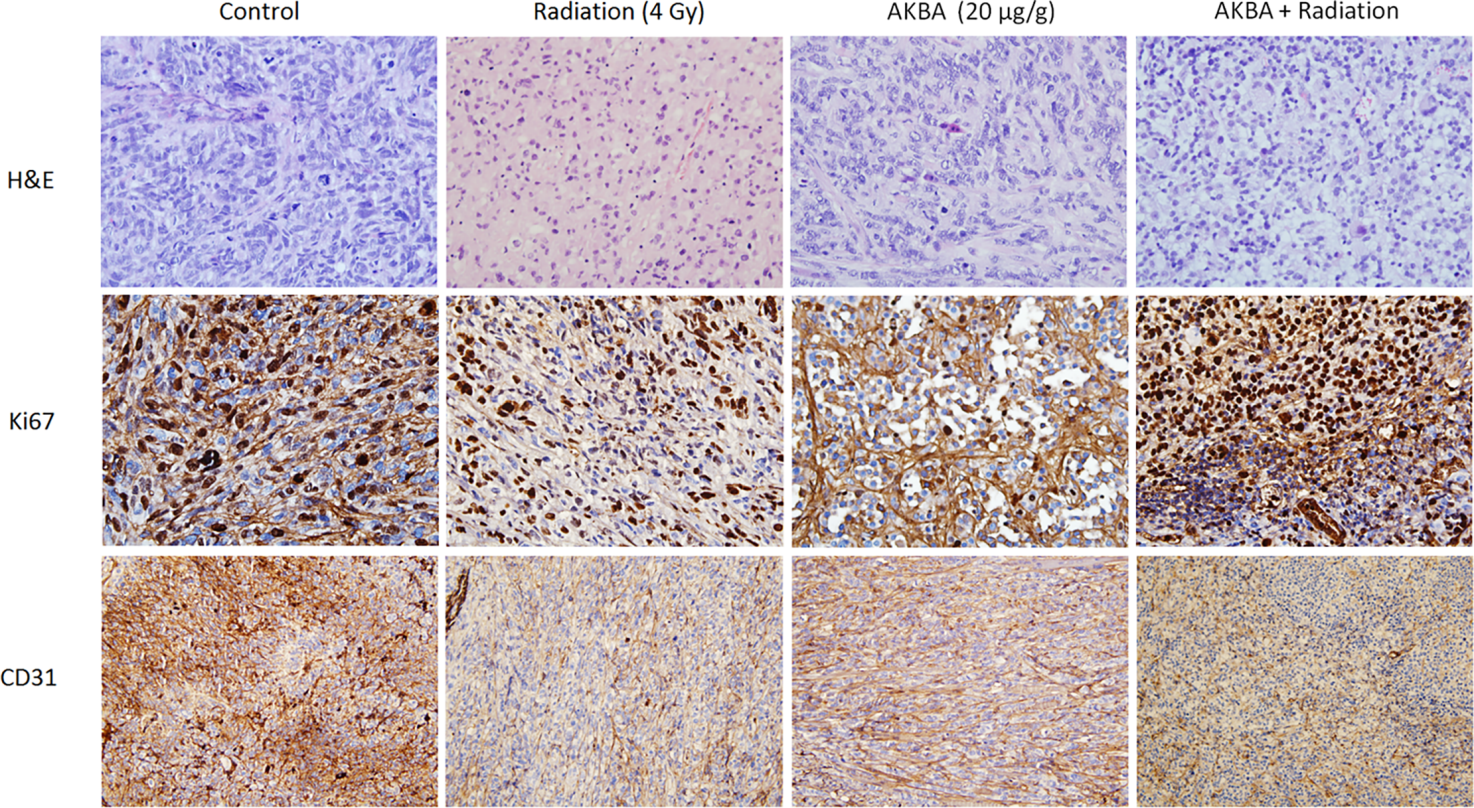
Effect of AKBA treatment on proliferation and intra-tumoral microvessel density in an ectopic GBM model. Formalin-fixed paraffin-embedded sections were stained immunohistochemically for Ki-67 (proliferation marker) and CD31 (endothelial marker). Representative microphotographs from each treatment group are shown (original magnification x400 –Ki-67, x200- CD31).

Proliferation is a key feature of tumor progression. Ki-67 is a human nuclear protein whose expression is strongly correlated with cancer cell proliferation. Immunohistochemical analysis of Ki-67 expression in the GBM tumors indicated that AKBA treatment inhibited U251 cell proliferation. Compared to controls, samples from the 20 μg/g AKBA-treated mice showed a substantial decrease in nuclear staining and in cell proliferation accompanied by an increase in necrotic regions (Fig 4). The number of Ki-67 positive cells was also markedly reduced in the group that received only radiation compared with the control group.

Pathological angiogenesis is a hallmark of cancer, and it plays an important role in proliferation as well as in the metastatic spread of cancer cells. In order to assess the effect of the tested treatments on intra-tumoral microvessel density, which is an indicator of new vessel growth, the expression of the endothelial cell-specific marker CD31 in the GBM tumor tissues was evaluated by immunohistochemical staining. As shown in Fig 4, CD31staining in the AKBA-treated tumors was significantly reduced, demonstrating anti-angiogenic activity of AKBA *in vivo*. A similar decrease in CD31 staining was observed following the combined AKBA and radiation treatment (Fig 4).

### Effect of AKBA and radiation on p53 and Bcl-2 expression in GBM tumors

**Fig 5:**
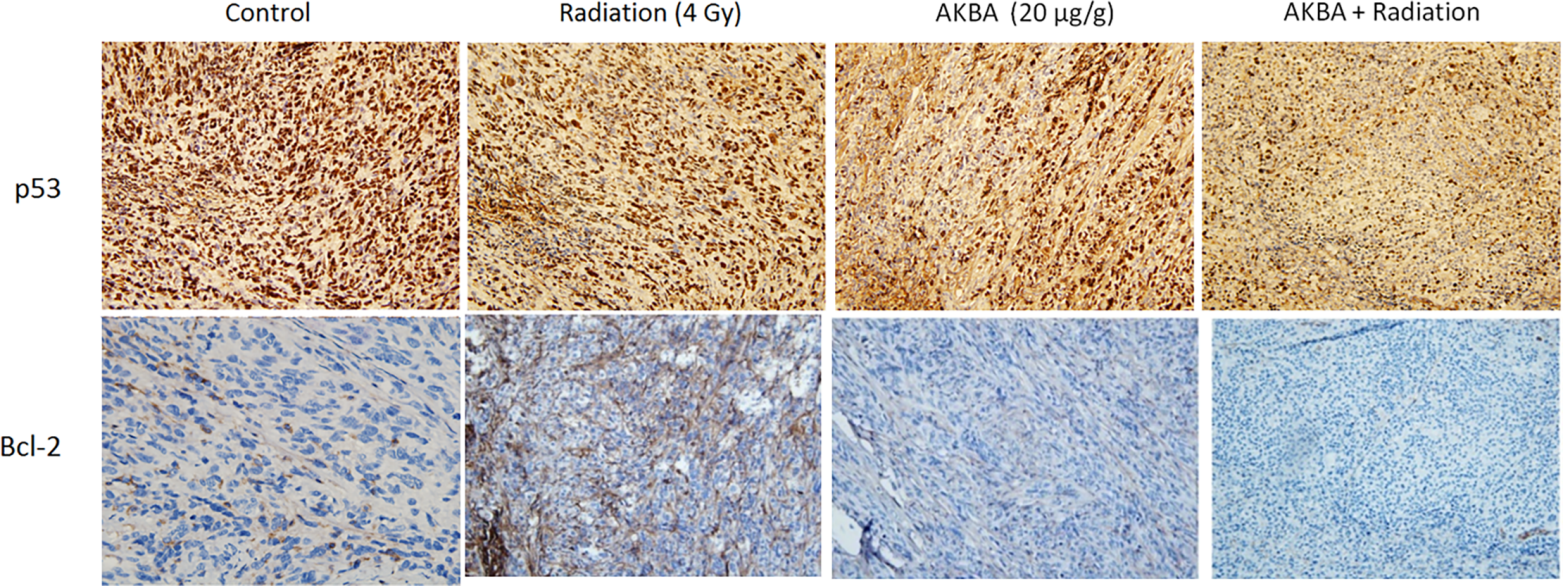
Effect of AKBA treatment on p53 and Bcl-2 expression in an ectopic GBM model. Formalin-fixed paraffin-embedded sections were stained immunohistochemically for p53 and Bcl-2. Representative microphotographs from each treatment group are shown (original magnification x200).

P53 gene expression was immunohistochemically detected by means of DO7 antibody, which identifies both the wild-type and the mutant-type p53 protein. Very strong nuclear staining was observed in the control samples, with almost 90% of the cells exhibiting stained nuclei (Fig 5). In contrast, the AKBA-treated samples as well as the samples from the combined treatment group showed a significant decrease in p53 nuclear staining (Fig 5).

We also investigated the effect of AKBA and/or radiation on the expression of anti- apoptotic Bcl-2 protein. As shown in Fig 5, AKBA induced a moderate down-regulation of Bcl-2 (cytoplasmic stain), whereas the combined treatment substantially decreased the expression of this anti-apoptotic protein. Radiation treatment alone had no impact on Bcl-2 expression.

### AKBA effect on NF-ĸB-related protein expression

**Fig 6:**
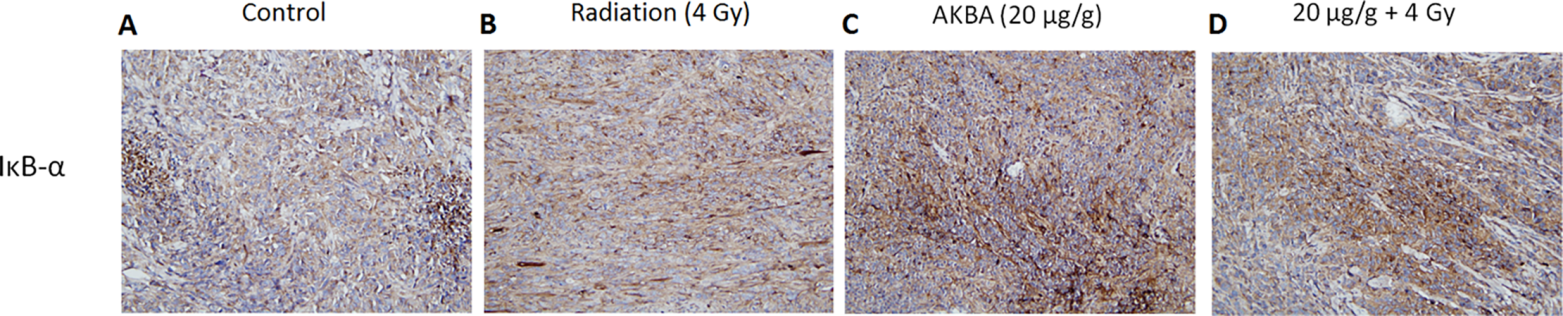
Effect of AKBA treatment on NF-ĸB-related protein expression in an ectopic GBM model. Formalin-fixed paraffin-embedded sections were stained immuno-histochemically for IĸB-α. Representative microphotographs from each treatment group are shown (original magnification x200).

We investigated also whether the effects of AKBA on tumor growth in mice were associated with expression of NF-kB-related proteins. IĸB-α is an inhibitor of NF-kB which masks the nuclear localization signals located on each NF-κB subunit to prevent their nuclear translocation. Following AKBA treatment, the tumor tissue showed an increase in IĸB-α staining (cytoplasmic) compared to sections from the control group (Fig 6). An increase in the staining was also found in certain areas of the tumor tissues from the combined treatment group, together with a marked increase of necrotic areas (Fig 6). Unstained areas of those samples coincided with the presence of fibrotic tissue surrounding the tumor and not with the tumor itself. No significant changes in NF-kB-p65 expression were detected following AKBA treatment alone or in combination with radiation (data not shown).

In this study, we demonstrated that AKBA exerts potent anti-proliferative activity and significantly inhibits both the survival of glioblastoma cells *in vitro* and the growth of tumors generated by these cells. AKBA induced apoptosis in glioblastoma cells which was associated with PARP cleavage. PARP is a zinc-dependent DNA-binding protein with a key role in DNA repair and its cleavage by caspase-3 is regarded as a specific marker for apoptosis. These findings are consistent with previous studies, showing that the main death mechanism induced by AKBA is apoptosis [6,8,13,32]. AKBA anti-tumor activity *in vivo* was associated with a decrease in the expression of Ki-67, a proliferation marker, as well as with a decrease in the expression of CD31, a marker for microvessel density. Vascularization provides the tumor supply of oxygen and nutrients and enables the removal of waste products. Thus, the inhibition of microvessel formation results in a decrease in tumor proliferation and metastatic spread. The ability of AKBA to suppress angiogenesis has been previously reported. In a study that investigated the effect of AKBA on prostate tumor xenograft mice, daily treatment with AKBA resulted in inhibited tumor angiogenesis [33]. This inhibition was correlated to a blockade of VEGFR2 activation and its multiple downstream signaling components [33]. Another study that was conducted on glioblastoma cells and explored how the kinetics of vascular normalization by VEGRR2 blockade governs tumor response to radiation demonstrated that VEGFR2 suppression can temporarily normalize tumor vessel structure, leading to improved vascular function and enhanced response to radiation therapy [34]. It follows, therefore, that AKBA’s anti-angiogenic activity could be employed to augment glioblastoma tumor susceptibility to radiation.

Mutations in the TP53 gene are frequent genetic alterations in human GBM. They are associated not only with the loss of tumor suppressor function, but also with a gain of function by switching on genes involved in the glioblastoma pathway [35]. Deregulation of the p53 pathway may also contribute to the treatment-resistant phenotype of GBM. In fact, p53 signaling was found to be altered in 64% of all GBMs [36], and 37% of them displayed mutations or homozygous deletions of the TP53 gene [37]. Our data showed that AKBA decreased the expression of p53 in ectopic GBM tumors derived from the p53-mutant glioma cell line U251. AKBA may also decreased tumor progression by the inhibition of a mutated p53 pathway.

Bcl-2 family proteins play key roles in cell death regulation, and they include both positive and negative regulators of apoptosis. Alterations in their expression and function contribute to the pathogenesis and progression of human cancers, including GBM. In particular, expression of the Bcl-2 family protein shifts towards an anti-apoptotic setting during progression from initial to recurrent GBM. Such a shift may contribute to glioma cell resistance to chemotherapeutic drugs and irradiation [38]. Bcl-2 is a pro-survival member which precludes the release of cytochrome-c from the mitochondria and the consequent activation of caspase cascade, thereby protecting the cells from apoptosis [39]. Irradiation was found to activate cell survival factors in glioblastoma cells and, specifically, to increase cellular levels of Bcl-2 and Bcl-x [40]. Down-regulation of the Bcl-2 protein enhanced the effect of radiation treatment on malignant glioma cells [41], whereas forced over-expression of Bcl-2 reportedly conferred only partial protection from irradiation [38]. It could be concluded, therefore, that combining radiation therapy with an adjuvant treatment that counteracts cell survival pathways could represent a valid strategy to enhance radio-sensitivity. The findings of the present study showed that the combined treatment by AKBA and ionizing radiation induced a substantial decrease in the expression of the anti-apoptotic protein Bcl-2. Such down-regulation could be one of the mechanisms underlying AKBA pro-apoptotic and radiosensitizing activity.

We demonstrated that NF-ĸB activation correlated to the pathogenesis of glioma. One of the mechanisms leading to aberrant activation of the NF-ĸB signaling pathway in glioblastoma is deletion of the NFKBIA gene that encodes IĸB-α. Loss of this key inhibitor results in a constitutive activation of NF-ĸB and is associated with disease progression, tumor recurrence, unfavorable therapy outcomes and short survival. A recent study showed that restoration of NFKBIA expression attenuated the malignant phenotype of cells cultured from tumors harboring the NFKBIA gene deletion. Increased expression of NFKBIA in those cells also sensitized them to temozolomide chemotherapy [42].

NF-ĸB activation is also linked to cellular resistance to chemotherapeutic drugs and to radiation treatment. Upon activation following various stimuli, such as irradiation, the NF-ĸB inhibitor IĸB-α is degraded through ubiquitin-mediated proteolysis. As a result, the heterodimer p50/p65 translocates to the nucleus where it acts as a transcription factor for a large number of proteins, a substantial number of which are well-described anti-apoptotic proteins. It has been reported that inhibition of NF-kB improves the apoptotic response to radiation therapy in glioblastoma cells. In particular, over-expression of IĸB-α in various malignant glioma cell lines increased their sensitivity to radiation compared to the parental cells that expressed low levels of IĸB-α protein [43]. Thus, by increasing the expression of IĸB-α, an NF-κB blockade could be combined with radiation therapy in order to increase its efficiency. Immunohistochemistry analysis in the current *in vivo* study showed a significant up-regulation of the NF-κB inhibitor IĸB-α following AKBA treatment, both in samples that received only AKBA and in samples that received the combined treatment. In light of the strong link between NF-κB activation, radioresistance and glioma pathogenesis, the ability of AKBA to down-regulate and modulate NF-κB signaling by increasing IĸB-α expression may be exploited to attenuate the glioblastoma cells’ malignant phenotype and, at the same time, increase cell susceptibility to radiation.

In the future AKBA could be a potential additional therapy for GBM. Since Kruger et al. [30] found very promising results demonstrating a higher BBB penetration of AKBA compared with that for KBA. Therefore we used AKBA in our study. In the future when AKBA will be used in clinical trials we should develop method to increase the lipid solubility of the drug, this may increase penetration across the BBB.

## Conclusions

In this study, we found that AKBA demonstrated anti-proliferative and apoptotic effect, and significantly inhibits survival of glioblastoma cell and the growth of tumors. Combination of AKBA with radiotherapy was found to inhibit factors which involved in cell death regulation, tumor progression and radioresistence, therefore it may serve as a novel approach for GBM patients.

## Supporting information

S1 Table Effect of AKBA, radiation and combined treatment on tumor growth in an ectopic GBM model. Tumor growth summary in excel file showing ANOVA statistic results.
(XLSX)
